# One-Week High-Dose β-Alanine Loading Improves World Tour Cyclists’ Time-Trial Performance

**DOI:** 10.3390/nu13082543

**Published:** 2021-07-25

**Authors:** Vicente Ávila-Gandía, Antonio Torregrosa-García, Silvia Pérez-Piñero, Raquel Ortolano, María Salud Abellán-Ruiz, F. Javier López-Román

**Affiliations:** 1 Sports Physiology Department, Faculty of Health Sciences, UCAM Universidad Católica San Antonio de Murcia, 30107 Murcia, Spain; vavila@ucam.edu (V.Á.-G.); atorregrosa@ucam.edu (A.T.-G.); rortolano@ucam.edu (R.O.); msabellan@ucam.edu (M.S.A.-R.); jlroman@ucam.edu (F.J.L.-R.); 2 Primary Care Research Group, Biomedical Research Institute of Murcia (IMIB-Arrixaca), 30120 Murcia, Spain

**Keywords:** β-alanine, sustained-release, time-trial, world tour cyclists, ergogenic aid

## Abstract

Supplementation with β-alanine is becoming a common practice in high-performance athletes. The purpose of the present study was to investigate the effects of a one-week high-dose β-alanine loading phase employing a sustained-release powder on preserving the time-trial performance capacity of world tour cyclists during overreaching training. Per day, 20 g of sustained-release β-alanine was administered during one week (7 days) of intensive team training camp in a randomised balanced placebo-controlled parallel trial design, with six participants in each β-alanine (BA) or placebo (PLA) group. A 10-min time trial (10′ TT) was carried out to analyse performance and biochemical variables. Anthropometry, paresthesia, and adverse event data were also collected. Power-based relative training load was quantified. Compared to placebo, the BA improved mean power (6.21%, 37.23 W; 95% CI: 3.98–70.48 W, *p* = 0.046), distance travelled (2.16%, *p* = 0.046) and total work (4.85%, *p* = 0.046) without differences in cadence (*p* = 0.506) or RPE. Lactate (*p* = 0.036) and anion gap (*p* = 0.047) were also higher in the BA group, without differences in pH or Bicarbonate. High daily and single doses were well tolerated. One-week high-dose β-alanine loading with a sustained-release powder blend can help attenuate 10′ TT performance losses of world tour cyclists due to intensive training.

## 1. Introduction

Intake of β-alanine (β-ala) has become popular in high-level athletes, becoming one of the world’s most popular supplements, and being one of the five currently endorsed by the International Olympic Committee [1]. Once inside the organism, it must be combined with L-histidine (L-his) to form a dipeptide called carnosine (Carn), which exerts the ergogenic effect associated with various physiological functions such as antioxidant activity, prevention of protein glycation reactions, increased sensitivity of calcium in muscle contraction, and a great capacity for intracellular buffering of hydrogen (H^+^) [2,3]. Supplementing with supraphysiological doses is one of the most effective methods of increasing muscle. Carn [3,4] is the limiting factor for acute β-ala intake the appearance of paresthesia: an uncomfortable itching or tingling sensation lasting approximately one hour [5] which can be avoided by administering smaller amounts throughout the day [6]. Carn elevation improves performance in high-intensity exercises in which increased muscle acidity (decreased muscle pH by an increased intramuscular H^+^) impairs muscle contractions by buffering these protons, prolonging the time of the onset of fatigue of bouts lasting between 30 s and 10 min [7]. In addition, Carn can increase the sensitivity of calcium in muscle myofibrils and seems to function as a kind of local H^+^/Ca^2+^ exchange pump for microdomains generated in the sarcolemma [3,8], characteristic of high-intensity climbing segments and sprints in professional cycling. Several weeks of daily β-ala supplementation (1.6–6.4 g in most studies) are required to significantly increase muscle carnosine and experience its physiological benefits [9], but no study has so far attempted higher daily doses to shorten this time span, due to the greater risk of triggering paresthesia. Sustained-release β-ala formulas can reduce this sensory side effect allowing higher single doses which, in theory, could reduce this minimum time required [10], but to date, no research has been carried out using a one-week high-dose loading strategy in competitive cyclists.

Professional world-tour road cycling is a sport with prodigious aerobic demands, but it has climbing segments and sprints intervals which force the rider to perform above its physical steady state, relying on the anaerobic glycolytic system for ATP renewal (producing an intramuscular pH decline by the H^+^ ions produced) which eventually reduces power during short-duration high-intensity bouts by muscle acidosis accumulation after repeated efforts in mountain ascents and sprints [11]. It is in these bouts, and especially in the time-trial (TT) stages, where supplementation with β-ala could help to maintain power output within each TT between races, but different outcomes were observed in trained or moderately trained cyclists [2] and further testing in highly trained cyclists is required. The high-intensity nature of competition, as well as a demanding schedule of international competition, frequently leads to overreaching. In this respect, β-ala could potentially help maintain training load without excessive fatigue, or increase training intensity [12], hypothetically helping during these physiologically overreaching situations, this requires initial trial testing.

In this study, we assessed a one-week high-dose β-ala loading phase as a short-term strategy to maintain high intensity cycling performance of highly trained World Tour cyclists after a week of intensive training camps. We hypothesised that power output would be improved in the time-trial by β-ala supplementation.

## 2. Materials and Methods

### 2.1. Subjects

Cyclists belonged to a Union Cycliste Internationale (UCI) World Team in the top 5 road category of elite male the previous year of the commencement of the study (2019), according to UCI’s ranking for team World Ranking officially published on its webpage [13].

### 2.2. Study Design

**Figure 1:**
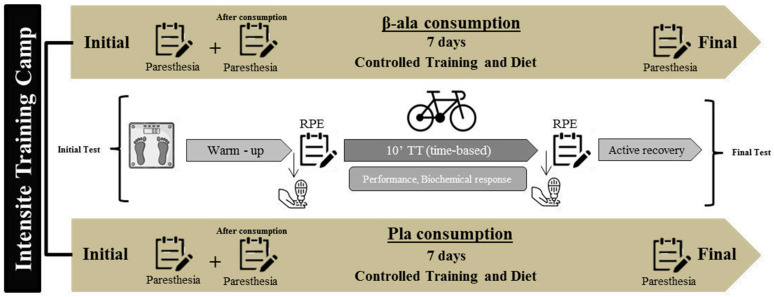
Graphical representation of the study design. RPE: Rating of Perceived Exertion.

A graphical representation of the study design is depicted in Figure 1. A double-blind, placebo-controlled, randomised, clinical trial was employed during a lapse of one intensive training camp week (7 days). A 10-minute time-trial was performed to quantify performance and physiological responses at the beginning and end of the intervention. Subjects were randomly assigned to a group of the 2 study arms: treatment group (BA) or control group (PL) and randomised using software (Epidat 4.2, Galicia, Spain). The research was carried out in the initial period of the training season (December 2019–August 2020), where the teams usually make training camps. The study was in agreement with the Declaration of Helsinki and was approved by the Clinical Research Ethics Committee of the Catholic University of Murcia (UCAM), study number CE012004 and Clinical trial ID NCT04427319 (June 2020).

Inclusion criteria were: (a) male professional active cyclist belonging to a UCI World Team; (b) starting the study in a well-rested condition: not performing intense training the day before. The exclusion criteria were: (a) chronic disease; (b) had suffered an injury which impeded training during the month before commencement of the study; (c) allergy to β-ala or any component of the placebo: wheat, soy, nuts (including peanuts), sesame or any of its by-products; (d) not understanding or refusing to sign the written consent prior the study; (e) previous β-ala supplementation within the two months before commencing the study; (f) use of any performance enhancer supplement 15 days before the study or during it.

Participants provided written informed consent prior to the commencement of the study, and could leave the study at any moment, with or without providing a reason and without further consequences.

### 2.3. Exercise Performance Test and Physiological Responses

A 10-min time-trial (10′ TT) was performed on a direct-drive electronically braked ergometer (Cyclus2, RBM elektronik-automation GmbH, Leipzig, Germany) using the official road bike model used for world tour competition. Subjects had to meet the following conditions: (1) refraining from intense physical exercise the day before; (2) being in a fasted state; (3) not consuming any ergogenic aid or drug treatment affecting performance. A 10-minute pre-competitive warm-up, followed by a passive 2-minute recovery was performed before the trial. During the trial, physical plate and sprockets remained fixed (plate × sprocket: 53 × 11), and participants had to change their virtual speeds through the ergometer control panel. Additionally, all data (power, pedal force, total work, speed and distance) except time were hidden from the display to blind subjects about their performance. Mean power output (MPO), peak power output, and distance covered were measured as performance variables. Total work, pedal force and average cadence were also recorded. Rate of perceived exertion (RPE) was measured by a modified 10-point Borg scale. After the test, cyclists pedalled for an additional three minutes at constant load (50 W) to recover.

Microcapillary blood samples were withdrawn one minute after the warm-up (during the passive recovery), and at the end of the 10′ TT (during the three-minute recovery), further analysed by a blood gas analyser (ABL90FLEX, Radiometer Medical APS, Copenhagen, Denmark). This test provided: partial pressure of O_2_ (pO_2_) and CO_2_ (pCO_2_), acid base equilibrium (ABE), bicarbonate (HCO_3_−), pH, anion gap and lactate.

### 2.4. Supplementation Loading Phase

The BA group consumed a sustained-release β-ala powder blend (BETAFOR3MAX^®^, Martinez Nieto S.A., Cartagena, Spain) for 7 days, in 4 daily intakes just after meals (breakfast, lunch, snack and dinner), leaving at least 4 h between each. Each single intake consisted of 5 g of β-ala, 37.5 mg of L-histidine and 12.5 mg of carnosine (20 g of β-alanine, 187.5 mg of L-histidine and 62.5 mg of carnosine per day in total). The pharmacokinetic response was previously studied, suggesting its suitability to provide high daily doses in a short-term strategy (Lydia de Salazar et al., submitted). We discarded carnosine to have an ergogenic effect different from its constituents β-ala and L-histidine, representing an additional donor of them. L-histidine on the other hand, can impact mental fatigue, but at a much higher daily dose (1.65 g daily for 2 weeks) [14].

Subjects started supplementation after the physical tests (just after the evening snack) and consumed the last intake after breakfast the same day of the final test (in total 7 days and 3 additional intakes, 31 servings). Total cumulative amount during the whole study was: 155 g of β-alanine, 1.45 g of L-histidine and 96.87 mg of carnosine. The batch employed (PI080120NID) was tested against doping substances by an external laboratory (Informed Sports LGS Supplement Screen service, LGC Group, Cambridgeshire, United Kingdom) and none were found (certificate of analysis number 3454). Additionally, the product was tested to for purity and label claim compliance as described elsewhere (Lydia de Salazar et al., submitted). The control group (PL) was provided a placebo (uncooked wheat semolina from Triticum durum) similar in appearance, instructed at the same dose by volume (one scoop), which provided a small amount of macronutrients (33 g of semolina per day; energy: 126 Kcal, protein: 3.8 g, carbohydrate: 23.1 g, of which sugar were only traces, fibre: 1.3 g, fat: 0.2 g). We assumed this placebo to lack any ergogenic effect. Both products were manufactured by the same company in sealed opaque containers weighed for compliance, and labelled for proper identification including participant number and trial code. To qualify for compliance, a maximum of 2 missed servings (representing a 6.45% of the total 31 servings) were allowed, checked by subtracting this quantity in grams from the total amount consumed. Subjects were followed-up after each intake, and to avoid cheating (emptying containers to pretend compliance) and to prevent serving shortage (in case of accidental spilling), additional quantity was included in containers.

### 2.5. Paresthesia Test

To check blinding of subjects, subjective symptoms of paresthesia were recorded in three occasions: firstly before the first intake of the product (Baseline), secondly after the first day of intake (T.1 First Day), and thirdly after the last day (day 7) of intake (T.2 Last Day). The questionnaire consisted of a quantitative question about intensity feelings, a qualitative question about type of sensory sensation and an empty box to report any other perceived sensation of side effect (see Paresthesia Test Document, Supplementary Material S1, which displays these sheets given to participants). Additionally, subjects were surveyed about their beliefs on which product they received after the study.

### 2.6. Diet and Body Weight

Subjects were given a nutrition factsheet designed by a nutritionist, and all followed the same diet guidelines depending on the training day. This contained information about the menu they had to eat on each meal (breakfast, lunch, snack and dinner), the foods they must choose (e.g., boiled vegetables, grilled meat, green beans…), and qualitative information about meal composition (e.g., carbohydrates with fibres, protein, low simple sugars, more fat). Additionally, they were instructed not to take snack a after long training and to avoid high amounts carbohydrate foods and simple sugars during recovery days, as suggested by the team’s nutrition specialist. Subjects ate at the lunch facilities of the host hotel which offered a buffet service and ate at the same time in presence of the nutrition specialist who prescribed the diet guidelines. and verified food intake. Prior to each physical test, anthropometric measurements of height (Seca 700, GmbH, Hamburg, Germany) and body weight (TANITA BC-420MA, Tanita Corporation, Tokyo, Japan) were performed.

### 2.7. Training Plan

All subjects were prescribed the same training plan during the week and recorded their training through their personal GPS bike computer (Garmin Edge 530, Garmin International, Olathe, KS, USA) to obtain training data and check compliance during the team camp. The training plan consisted mainly of one or two outdoor cycling routes of hilly courses around the area of Calpe (Alicante, Spain) performed in the morning and/or evening. Two days (day 3 and day 7) consisted of regenerative cycling workouts at low intensity. All participants went cycling together at the same time of the day, thus homogenising the external conditions (such as wind) of each outdoor training workout. Power data were recorded through left and right power pedals (Garmin Vector 3, Garmin International, Olathe, KS, USA) which were previously installed and calibrated by a specialised mechanic who adjusted the torque of its screws to 34 N-m as specified by manufacturer.

### 2.8. Training Status and Critical Power

An incremental cycling test to exhaustion performed one week before was employed to assess training status of participants and determine each volunteer’s critical power to quantify the individualised training load. Previous studies showed a good correlation between power output at the respiratory compensation point or ventilatory threshold 2 (WVT2) and the critical power [15,16,17], which demarcate the heavy from severe exercise intensity domains. Methods, equipment and repeatability measures were the same as described elsewhere [18], but fixing was without wheels, pedalling always with road cycling shoes, and the incremental ramp test consisted of a 4 min warm-up at 100 W, followed by ramp test with 5 W increments every 12 s (25 W per minute). Ventilatory aerobic and anaerobic threshold were interpreted according to the three-phase model by ventilatory equivalents (VE). VT2 was set as the intersection point between the carbon dioxide ventilatory equivalent (VE/VCO_2_) and the oxygen ventilatory equivalent (VE/VO_2_) against time. Power output at the ventilatory threshold 2 (WVT2) was adopted as the individual critical power (CP) and the rest of variables to determine training status expressed as relative maximum aerobic consumption (VO_2max_).

### 2.9. Training Load Quantification

Training load was calculated as the daily average during the training camp week. Power-based quantification was performed according to the Skibba’s Bike Score model [19,20,21]. We decided to employ this method instead of the functional threshold power (FTP) due to a closer correlation to critical power [22] both in magnitude and placement to ventilatory threshold 2 [23,24]. Raw data extracted from the GPS bike computer were introduced in the cycling software (Golden Cheetah training software, version 3.5, available at www.goldencheetah.org, accessed on 14 October 2020) which performed the calculations based on each subject’s critical power. Bike Score (expressed in arbitrary units) is dependent on the relative intensity (RI) of a workout, which is multiplied by the normalised work (in Joules) and multiplied by 100. The RI is the division of xPower (normalised power computed as 25 s exponentially-weighed moving average) by the critical power, thus representing an intensity index above a theoretical steady-state threshold. The normalised work is the result of xPower (normalised power) multiplied by the duration of the workout in seconds, which represents the total energy spent during a workout.

### 2.10. Statistical Analysis

The per-protocol (PP) data set was analysed, that is, all participants who completed the study period. Continuous variables are expressed as mean and ±standard error of the mean (SEM). Data analysis included the Student’s t-test for comparison of quantitative variables between the study groups at baseline. Normality was tested by the Kolmogorov–Smirnoff test and homoscedasticity by the Levene test.

To analyse the differences between the groups in the evolution of the different variables, a parametric test such as an analysis of variance (ANOVA) for repeated measures was carried out, with time (initial test and final test) as the within subject factor, and intervention (BA and PL) as between-subject factor (for the post hoc analysis, Bonferroni tests were carried out) to compare the variation experienced by the variables during the week consumption. The sample size was calculated according to the mean power during a maximal 10′ TT test. Considering a standard deviation of mean power of 30.4 W [25], for a precision of 35 W with alpha risk of 5% and statistical power of 80%, 9 subjects in each group were needed, increasing to 10 subjects per group assuming a 10% loss to follow-up. Sample size was determined by the availability of cyclists’ professional schedule. Statistical analysis was performed using software (SPSS version 21.0, Chicago, IL, USA) and data visualisation using RStudio (version 1.1.4, available at https://www.rstudio.com/, accessed on 21 October 2020) with R programing language (version 3.4.4, available at https://www.r-project.org/, accessed on 21 October 2020) plotted by the ggplot2 package (version 2.2.1, available at https://ggplot2.tidyverse.org, accessed on 21 October 2020).

## 3. Results

### 3.1. Flow Chart and Participant Characteristics

**Figure 2:**
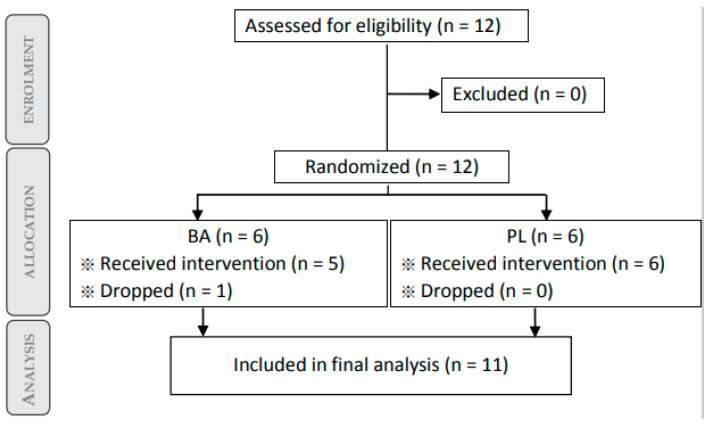
Participant flow chart.

From the twenty subjects calculated from sample size calculation, twelve world tour cyclists were initially recruited, and eleven were included in the final analysis (Figure 2). One subject from the BA group dropped from the study due to a race he had to attend in another country which affected his training load and made him unavailable in the second visit (final measurement). More details about participant characteristics can be found in Table 1.

### 3.2. 10′ TT Performance Analysis

**Figure 3:**
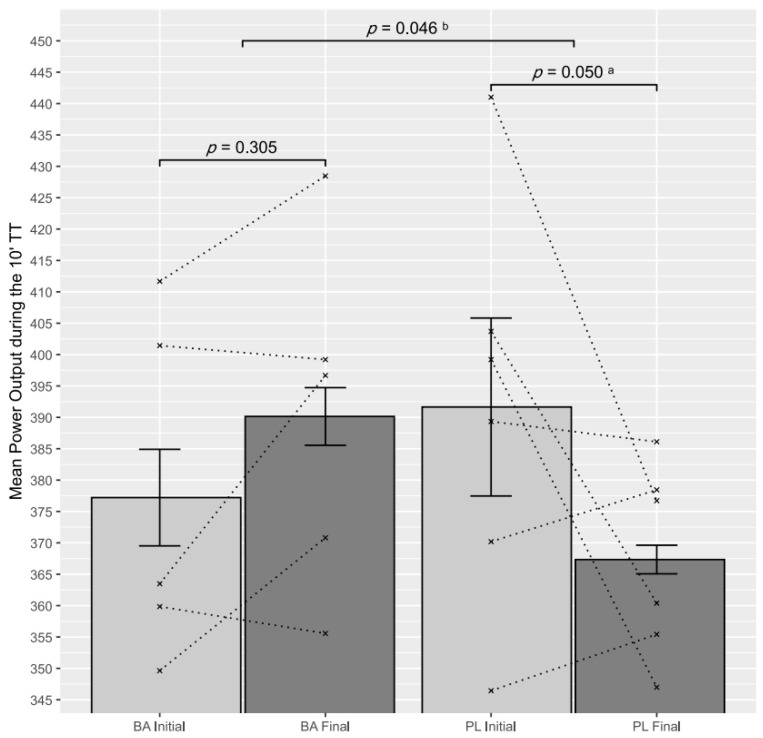
Mean power before and after the 10′ TT. ^a^: Significant Intragroup difference. ^b^: Significant time × group difference.

At baseline, no measures of physiology or performance differed between groups (Table 1) or the comparison of mean power output before supplementation (*p* = 0.449). Mean power output was a 6.21% higher (37.23 W; 95% CI: 3.98–70.48 W) in BA than in PL (*p* = 0.046, Figure 3 and Table 2) in parallel with a 2.16% increase in distance travelled (*p* = 0.046, Table 2) and a 4.85% in total work (*p* = 0.046, Table 2) after intervention. Intra-group comparison showed that the PL significantly reduced the mean power (−6.20%, −24.30 W; 95% CI: −48.88–0.00 W, *p* = 0.050), while the BA group preserved it (3.43%, 12.92 W; 95% CI: −13.99–39.85 W, *p* = 0.305). Average cadence and mean pedal force were not significantly different on both occasions within both groups, and when compared one to each other (Table 2). All tests qualified as maximal based on the reported ratings in the Borg scale (BA: 10 ± 0, *p* = 1; PL: 10 ± 0, *p* = 1).

Data are presented as mean and standard error of the mean at baseline and after 7 days of product and placebo consumption. The between-group *p*-value in the ANOVA for repeated measures with two study factors (time × group) is also reported. The within-group *p*-value (baseline and final) is also reported.

### 3.3. 10′ TT Physiological Responses

Lactate (*p* = 0.036) and anion gap (*p* = 0.047) were significantly increased in BA compared to placebo after treatment (Table 3). No other variables were different when comparing both groups or when comparing changes within each group before and after intervention. Lactate, pH, bicarbonate, and anion gap were significantly modified after the 10′ TT within groups on both occasions (*p* < 0.050), which suggests that the trial load was similar on both days.

### 3.4. Compliance and Paresthesia Test

All subjects consumed both the β-ala powder blend or placebo at the indicated doses without significant differences in the compliance control. No side effects or paresthesia were reported. From the eleven subjects, eight believed they consumed the experimental product (four from the BA group, and four from the PLA group), while one believed belonging to the control product (actually in the PLA group) and two doubted (one from BA and one from PLA). Thus, subjects which provided an answer were proportionally correct or incorrect in their guessing (five out of twelve, or 41.67% of the times).

### 3.5. Training Load and Body Weight

All subjects completed each workout of the training plan, described in detail for the whole week in Table 4 and per each day in Table 5. No significant differences were found in any quantitative metric of the training performed (moving time, distance, mean power, normalised power, relative intensity, average speed, elevation gain and total work performed) or relative training load (Bike Score). The relative intensity of regenerative cycling workouts (day 3 and 7) displays lower scores (PL = 0.50 and 0.53; BA = 0.55 and 0.53 respectively, Table 5) than workout days (PL = 0.72; BA = 0.74, Table 4). Body weight also remained similar within groups in the initial (PL= 67.2 ± 7.5; BA = 69.8 ± 4.1 kg) and final test (PL = 67.2 ± 7.6; BA = 69.3 ± 5.3 kg).

Values are presented as mean with standard error of the mean (between brackets). Bike Score (relative training load) is presented in arbitrary units (AU). The *p*-value between groups in the ANOVA for the mean of the 7 training days (entire week) and the 5 days of intense training is reported.

## 4. Discussion

The novel finding of the present study is that a short-term high-dose β-ala loading improved high-intensity cycling capacity and distance covered of elite cyclists engaged in an intensive training plan compared to a placebo. Maintaining performance during consecutive high-demanding stages can represent a competitive advantage for β-ala consumers competing at World Tour time-trial races, since small performance improvements can be considered as meaningful [26]. Our results are promising in this respect, although require further confirmation in trials with larger sample sizes (which may also yield a more accurate estimate of the size effect) and confirm generalizability of these findings in other highly trained cyclists and competition formats. The sample size recruited was small due to the low availability of these elite professional cyclists and imposed a limitation in the current study. To minimise the effect of other confounders, we established the criteria of maximum rate of perceived exertion (10 in the modified Borg scale) after each 10′ TT. Furthermore, when evaluating studies on β-alanine in athletes, the following three factors should be considered: the time-trial employed, the training status of the sample, and the total cumulative dose of β-ala provided as treatment (total grams during the whole study). Previous studies with β-ala in trained cyclists using time-trial tests observed that the greatest improvements were obtained in 4 km TT tests [25]. The sample physical conditioning difference makes inappropriate performance or magnitude comparison with the current study, and against the only study we found, employing a 10-min TT (since it was performed after a 110 min running simulation [27]).

In the present study, β-ala helped maintain performance following a period of increased training intensity. Overreaching is common at various points during the season in competitive cyclists, in response to increased volumes of training or competition [28,29] which ultimately reduces performance capacity [30]. To the best of our knowledge, only one study tested short-duration β-ala on males during an intensive training camp (last 9 days, 135 g of β-ala along 30 days) which did not improve anaerobic cycling capacity (Wingate test), but fatigue feelings [31], in line with another study on females which did not mitigate a reduction in high-intensity intermittent exercise capacity (YoYo and sprint test) during this preparatory period [32]. Both studies, together with this study, tested short-duration β-ala to help maintain performance when athletes are subject to a highly demanding physiological load, as is the case in intensive training camps or consecutive races during a professional athlete season.

In the current study, the high energy demand in the TT probably was satisfied by the anaerobic lactic glycolysis, consequently increasing intra-muscular H^+^ and lactate, which causes a deleterious state for the maintenance of muscle contraction. Increased H^+^ production is partly buffered inside the muscle by carnosine ionic microdomains, which deposits H^+^/Ca^2+^ in the cytoplasm to regulate intracellular pH, partly expelling the remaining H^+^ to systemic blood [3]. Previous studies with trained cyclists performing time-trials [25,27,33] or after 1-h of moderate intensity cycling [34] did not observe a difference in lactate or blood pH by β-ala. On the other hand, athletes engaged in high-intensity interval training yielded higher lactate values compared to placebo after these efforts [35,36] in line with our results. However, the buffering capacity of muscles did not change in these studies in spite of an increased muscle carnosine, suggesting an improved muscle force in short-duration high-intensity movements by acting on neuromuscular fatigue. In the current study, as the training week progressed, cyclists probably accumulated tiredness and fatigue characteristic of these intensive training camps [30,32], and β-ala helped to maintain mean power due to a preservation of the muscle buffering systems, action on central fatigue, or a combination of both. Recent studies in elite endurance athletes, sprinters and combat sports, suggest metabolic adaptation benefits (lower ammonia concentrations after exercise) during training periods [37,38] which may be an additional benefit of short-duration high-dose β-ala in lack of a visible performance improvement, in addition to a performance benefit.

In this study, we employed the highest single dose of β-ala (5 g per single dose), and daily dose (20 g daily) reported to date. The limiting factor in employing high single doses is the appearance of paresthesia, present in a large number of studies [6]. The product employed is a novel sustained-release β-ala powder blend, which enabled such high doses with no paresthesia in most subjects. Higher daily doses can reduce the loading period, increasing carnosine stores in shorter times. In the present study we employed a total cumulative amount of 155 g in a single (loading) week, which is a short time compared to other studies. The amount is within the range of the majority of the studies reviewed with cyclists, which supplied approximately 130–200 g, over 4–8 weeks, with variable results [2], but in the present study, 155 g of β-ala was sufficient to elicit benefits. This is encouraging, since such high rate loading protocols can enable short-term carnosine loading, which is a prevailing handicap in the study and usage of β-ala. However, as a limitation of the study, actual increases in muscle carnosine were not directly measured (by muscle biopsy or magnetic resonance spectrometry) and should be verified in future studies employing this loading protocols in professional cyclists. We expect that carnosine stores increased based on the bioavailability of the sustained-release power (de Salazar et al., submitted), the kinetic model proposed by Spelnikov and Harris [39], and the potential of trained muscles to obtain higher concentrations of carnosine [40].

Another limitation of the study is that we could not completely discard an instantaneous effect of the experimental product, given that it was the first time that 5 g of β-ala were provided in a single dose, and the scarcity of single-dose trials. When exploring the effects of informed (unblinded) vs. non-informed (blinded) β-ala supplementation, a possibly likely increase in mean power output (inconclusive for performance enhancement) was reported [41], while trained female cyclists (considered to be more sensitive to carnosine increases according to article’s authors) did not improved mean power during a Wingate test [42]. It seems unlikely that a single dose could increase carnosine enough to improve intramuscular buffering, but cyclists’ beliefs in an effect may had influence the maximal effort putted in the 10’ TT. Our results show that most cyclists believed they were receiving β-alanine independently of the treatment (four out of five in BA, and four out of six in PLA), balancing this possible influence.

High-dose β-ala loading may represent an effective short-term strategy to preserve performance of world tour cyclists racing in short time-trial courses during physiologically demanding stages. Future research may consider short-term high-dose loading phases as a feasible strategy to preserve cycling capacity in short time-trial stages of competitive elite athletes during physically demanding periods. Further studies with sustained-release formulas employing this high-dose, short-term loading are required to quantify actual muscle carnosine increases in professional cyclists.

## 5. Conclusions

One-week high-dose β-alanine loading with a sustained-release powder blend helped attenuate performance losses due to intensive training of world tour cyclists during a maximal 10′ TT test. Future research may consider short-term high-dose loading phases as a feasible strategy to preserve cycling capacity in short time-trial stages of competitive elite athletes during physically demanding periods.

## Figures and Tables

**Table 1:** World tour cyclist physical characteristics. Values are presented as mean with standard error of the mean inside brackets.

	PL	BA	Total	*p*-Value
Age (years)	25.3 (1.4)	25.7 (0.9)	25.5 (0.8)	0.841
Height (cm)	179.5 (3.2)	181.5 (1.5)	180.5 (1.7)	0.587
Weight (kg)	67.2 (3.1)	69.8 (1.7)	68.5 (1.7)	0.476
VO_2max_ (mL/kg/min)	68.0 (2.4)	67.6 (1.6)	67.8 (1.4)	0.897

PL (control group); BA (treatment group); *p*-value between-group of treatment.

**Table 2:** Performance analysis of the 10′ TT. Values are presented as mean with standard error of the mean between brackets. AUC: Area under the curve (calculated by trapezoidal rule). rpm: revolutions per minute. RPE: rate of perceived exhertion.

	Group	Initial Test	Final Test	Intra-Group *p*-Value	Product × Time *p*-Value	Power (1-β)
Mean Power (W)	PL	391.1 (12.3)	367.3 (9.0)	0.050 ^a^	0.046 ^b^	0.533
	BA	377.2 (13.5)	390.1 (9.8)	0.305		
Distance Tavelled (m)	PL	7379 (84)	7211 (61)	0.050 ^a^	0.046 ^b^	0.551
	BA	7276 (92)	7367 (67)	0.299		
Total Work (J)	PL	235.0 (7.4)	220.3 (5.4)	0.050 ^a^	0.046 ^b^	0.544
	BA	226.2 (8.1)	234.0 (5.9)	0.305		
Work AUC (J × m)	PL	70,520 (2212)	66,761 (1795)	0.122	0.050 ^b^	0.597
	BA	68,710 (2423)	72,204 (1966)	0.182		
Power AUC (W × m)	PL	214,162 (7206)	191,539 (5171)	0.005 ^a^	0.037 ^b^	0.557
	BA	204,465 (7893)	204,068 (5665)	0.954		
Average Cadence (rpm)	PL	106.6 (2.2)	107.4 (2.5)	0.704	0.506	0.138
	BA	106.6 (2.4)	105.4 (2.7)	0.577		
RPE	PL	10 (0)	10 (0)	1	1	1
	BA	10 (0)	10 (0)	1		
Mean Pedal Force (N)	PL	266.2 (13.9)	241.4 (14.0)	0.086	0.260	0.233
	BA	263.2 (15.2)	261.4 (15.4)	0.900		

PL (control group); BA (treatment group); The *p*-value between groups in ANOVA for repeated measures with two study factors (time × group) is reported. Intragroup *p*-value (initial and final) is also reported. ^a^: Significant Intragroup difference. ^b^: Significant time × group difference.

**Table 3:** Physiological responses: microcapillary blood biochemical analysis. Values are presented as mean with standard error of the mean (between brackets).

	Group	Initial Test		Final Tesst		Intra-Group *p*-Value	Product × Time *p*-Value
		Basal	Post Test	Basal	Post Test		
Lactate (mmol/L)	PL	1.9 (0.6)	12.2 (3.5) ^a^	1.9 (0.4)	10.9 (4.4) ^a^	0.208	0.036 ^b^
	BA	1.7 (0.6)	11.4 (2.4) ^a^	1.8 (0.4)	13.8 (2.6) ^a^	0.060	
Anion gap (mEq/L)	PL	8.4 (0.6)	20.4 (1.3) ^a^	9.1 (0.6)	19.2 (1.5) ^a^	0.182	0.047 ^b^
	BA	8.6 (0.6)	21.0 (1.4) ^a^	9.2 (0.6)	24.2 (1.6) ^a^	0.107	
pH	PL	7.400 (0.008)	7.233 (0.019) ^a^	7.404 (0.007)	7.253 (0.036) ^a^	0.510	0.606
	BA	7.416 (0.009)	7.225 (0.020) ^a^	7.431 (0.008)	7.238 (0.039) ^a^	0.923	
Bicarbonate (mmol/L)	PL	24.9 (0.5)	15.1 (0.6) ^a^	25.0 (0.6)	15.9 (1.2) ^a^	0.495	0.576
	BA	25.1 (0.6)	14.7 (0.6) ^a^	25.0 (0.6)	14.5 (1.3) ^a^	0.894	
ABE (mEq/L)	PL	0.7 (0.6)	−12.4 (0.9) ^a^	0.9 (0.7)	−11.4 (0.7) ^a^	0.536	0.601
	BA	0.9 (0.7)	−13.2 (1.0) ^a^	0.8 (0.7)	−13.5 (1.9) ^a^	0.887	
pCO_2_ (mm of Hg)	PL	41.5 (1.1)	33.0 (1.1) ^a^	41.3 (1.2)	32.6 (1.0) ^a^	0.975	0.868
	BA	39.7 (1.2)	31.4 (1.2) ^a^	37.2 (1.3)	28.4 (1.1) ^a^	0.801	
pO_2_ (mm of Hg)	PL	66.4 (2.3)	78.3 (2.8) ^a^	70.4 (4.5)	85.1 (3.2) ^a^	0.499	0.132
	BA	68.8 (2.6)	85.7 (3.0) ^a^	79.8 (5.0)	89.8 (3.5)	0.143	

PL (control group); BA (treatment group); ABE: Acid base equilibrium; The *p*-value between groups in ANOVA for repeated measures with two study factors (time × group) is reported. Intragroup *p*-value (initial and final) is also reported. ^a^: Significant Intragroup difference. ^b^: Significant time × group difference.

**Table 4:** Descriptive analysis of the mean daily load of all workouts during the one-week training camp and the mean of the 5 training days excluding regenerative workouts.

Metric	Average 7 Days Training			Average 5 Days Intense Training			*p*-Value
	PL	BA	Total	PL	BA	Total	
Moving time (hours)	3.88 (0.10)	3.91 (0.10)	3.89 (0.05)	4.78 (0.30)	4.85 (0.19)	4.81 (0.08)	0.766
Distance (km)	122.7 (2.6)	124.0 (2.8)	123.3 (1.8)	152.5 (5.7)	155.5 (2.7)	153.9 (2.8)	0.754
Mean Power (W)	192.0 (7.6)	177.8 (8.4)	185.5 (5.8)	203.4 (3.0)	187.3 (6.1)	196.1 (6.8)	0.242
Normalized power (W)	239.5 (7.5)	240.2 (8.3)	239.8 (5.3)	257.13 (3.99)	259.92 (3.8)	258.4 (5.7)	0.948
Total work (kJ)	2718.9 (110.8)	2748.0 (121.4)	2732.0 (78.0)	3459.9 (197.4)	3503.1 (148.0)	3479.0 (106.0)	0.863
Relative Intensity (RI)	0.66 (0.02)	0.69 (0.02)	0.67 (0.13)	0.72 (0.01)	0.74 (0.01)	0.73 (0.01)	0.333
Bike Score (AU)	317.3 (32.3)	351.6 (30.5)	318.8 (32.5)	275.6 (25.8)	313.0 (23.8)	292.6 (23.5)	0.414

PL (control group); BA (treatment group).

**Table 5:** Description of training schedule per day. Values are presented as mean with standard error of the mean (between brackets).

		Day 1	Day 2	Day 3	Day 4	Day 5	Day 6	Day 7
Moving time (hours)	PL	5.16 (0.11)	5.50 (0.01)	1.74 (0.14)	4.55 (0.12)	4.80 (0.56)	3.90 (0.22)	1.47 (0.06)
	BA	4.94 (0.11)	5.50 (0.03)	1.57 (0.02)	4.68 (0.11)	4.80 (0.43)	4.33 (0.36)	1.53 (0.03)
	TOTAL	5.06 (0.08)	5.50 (0.01)	1.66 (0.08)	4.61 (0.08)	4.80 (0.35)	4.10 (0.20)	1.50 (0.03)
Distance (km)	PL	162.5 (3.4)	164.1 (0.4)	54.7 (3.8)	146.6 (4.9)	156.2 (20.0)	133.1 (7.5)	41.9 (2.3)
	BA	156.4 (3.5)	164.4 (0.2)	46.1 (2.0)	151.5 (3.8)	157.0 (13.6)	148.4 (14.8)	43.9 (0.3)
	TOTAL	159.7 (2.5)	164.2 (0.2)	50.8 (2.5)	148.8 (3.1)	156.5 (11.9)	140.0 (7.8)	42.8 (1.2)
Total work (kJ)	PL	3537.8 (104.1)	3984.3 (119.3)	898.7 (131.4)	3338.3 (172.9)	3648.3 (506.4)	2790.7 (199.0)	833.8 (36.9)
	BA	3456.6 (67.5)	3936.2 (85.3)	918.8 (52.9)	3497.4 (171.0)	3609.0 (344.5)	3016.4 (281.5)	801.6 (26.6)
	TOTAL	3500.9 (62.8)	3962.5 (72.6)	907.8 (72.3)	3410.6 (118.8)	3630.5 (302.6)	2893.3 (162.7)	819.2 (22.9)
Mean Power (W)	PL	195.7 (7.3)	207.3 (7.7)	165.0 (11.0)	206.3 (8.2)	210.7 (11.6)	197.2 (11.0)	161.7 (8.0)
	BA	165.0 (17.5)	198.4 (3.5)	163.0 (6.8)	190.2 (18.0)	185.6 (17.2)	197.4 (4.7)	144.8 (2.6)
	TOTAL	181.7 (9.7)	203.3 (4.5)	164.1 (6.4)	199.0 (9.2)	199.3 (10.3)	197.3 (6.1)	154.0 (5.1)
Normalized power (W)	PL	254.2 (8.7)	269.8 (7.7)	196.7 (12.2)	255.7 (8.9)	260.5 (14.0)	245.5 (13.9)	193.8 (7.3)
	BA	258.0 (6.7)	271.6 (6.0)	195.0 (4.0)	262.6 (8.4)	259.0 (13.6)	248.4 (3.6)	186.8 (3.7)
	TOTAL	255.9 (5.4)	270.6 (4.8)	195.9 (6.6)	258.8 (6.0)	259.8 (9.3)	246.8 (7.4)	190.6 (4.3)
Relative Intensity (RI)	PL	0.71 (0.02)	0.75 (0.02)	0.50 (0.05)	0.71 (0.02)	0.72 (0.03)	0.68 (0.03)	0.54 (0.02)
	BA	0.73 (0.02)	0.78 (0.01)	0.55 (0.02)	0.75 (0.02)	0.74 (0.04)	0.71 (0.01)	0.53 (0.01)
	TOTAL	0.72 (0.01)	0.76 (0.01)	0.52 (0.03)	0.73 (0.02)	0.73 (0.03)	0.70 (0.02)	0.54 (0.01)

PL (control group); BA (treatment group).

## Data Availability

Data availability statement has been adapted to meet journal’s policy.

## Supplementary Materials

The following are available online at https://www.mdpi.com/article/10.3390/nu13082543/s1, Supplementary Material S1: paresthesia test document.docx, which displays the sheets given to participants.
