# Supplementary material for: One-Week High-Dose β-Alanine Loading Improves World Tour Cyclists’ Time-Trial Performance
Source: Nutrients. 2021 Jul 25;13(8):2543. doi: 10.3390/nu13082543 (PMC8401416; doi:10.3390/nu13082543)
Supplement: Supplementary file 1 [file nutrients-13-02543-s001.zip › nutrients-1277412-supplementary.pdf]

A RANDOMISED DOUBLE-BLIND CLINICAL TRIAL TO ANALYSE THE EFFICACY OF SHORT-TERM β-ALANINE SUPLEMENTATION IN PERFORMANCE OUTCOMES OF ROAD PROFFESIONAL ENDURANCE CYCLISTS

DATE: 23 / 01 / 2020 **CODE:** ___ ___

SENSATION INTENSITY: VISUAL ANALOGUE SCALE (VAS)

Mark a vertical line according to closest perceived intensity **at this moment**. An absence of any unusual sensation corresponds to *No unusual sensation* and a mark of 0 and should coincide with the vertical line to the left and *Most intense sensation* corresponds to a mark of 10.

No unusual sensation |————————————————————| **Most intense sensation**

QUALITATIVE DESCRIPTION

From the following words, please mark or circle the ones that better describes the current sensation (if no sensation leave it blank).

Pins-and-needles
Itching
Tickling
Numbness
Shiver
Pain

Other:

____

____

____

Please describe in detail any other side effect of feeling not previously mentioned.

BASELINE **PARESTHESIA TEST**

A RANDOMISED DOUBLE-BLIND CLINICAL TRIAL TO ANALYSE THE EFFICACY OF SHORT-TERM β-ALANINE SUPLEMENTATION IN PERFORMANCE OUTCOMES OF ROAD PROFFESIONAL ENDURANCE CYCLISTS

DATE: 24 / 01 / 2020 **CODE:** ___ ___

SENSATION INTENSITY: VISUAL ANALOGUE SCALE (VAS)

Mark a vertical line according to the **highest intensity perceived during the first day**. An absence of any unusual sensation corresponds to *No unusual sensation* and a mark of 0 and should coincide with the vertical line to the left and *Most intense sensation* corresponds to a mark of 10.

No unusual sensation |————————————————| **Most intense sensation**

QUALITATIVE DESCRIPTION

From the following words, please mark or circle the ones that better describes the current sensation (if no sensation leave it blank).

Pins-and-needles Itching

Tickling Numbness

Shiver Pain

Other:

____

____

____

Please describe in detail any other side effect of feeling not previously mentioned.

T.1 FIRST DAY **PARESTHESIA TEST**

A RANDOMISED DOUBLE-BLIND CLINICAL TRIAL TO ANALYSE THE EFFICACY OF SHORT-TERM β-ALANINE SUPLEMENTATION IN PERFORMANCE OUTCOMES OF ROAD PROFFESIONAL ENDURANCE CYCLISTS

DATE: 31 / 01 / 2020 **CODE:** ___ ___

SENSATION INTENSITY: VISUAL ANALOGUE SCALE (VAS)

Mark a vertical line according to the **highest intensity perceived during the last day**. An absence of any unusual sensation corresponds to *No unusual sensation* and a mark of 0 and should coincide with the vertical line to the left and *Most intense sensation* corresponds to a mark of 10.

No unusual sensation |————————————————| **Most intense sensation**

QUALITATIVE DESCRIPTION

From the following words, please mark or circle the ones that better describes the current sensation (if no sensation leave it blank).

Pins-and-needles Itching

Tickling Numbness

Shiver Pain

Other:

____

____

____

Please describe in detail any other side effect of feeling not previously mentioned.

T.2 LAST DAY **PARESTHESIA TEST**
